# Optimal dose and safety of molnupiravir in patients with early SARS-CoV-2: a Phase I, open-label, dose-escalating, randomized controlled study

**DOI:** 10.1093/jac/dkab318

**Published:** 2021-08-27

**Authors:** Saye H Khoo, Richard Fitzgerald, Thomas Fletcher, Sean Ewings, Thomas Jaki, Rebecca Lyon, Nichola Downs, Lauren Walker, Olana Tansley-Hancock, William Greenhalf, Christie Woods, Helen Reynolds, Ellice Marwood, Pavel Mozgunov, Emily Adams, Katie Bullock, Wayne Holman, Marcin D Bula, Jennifer L Gibney, Geoffrey Saunders, Andrea Corkhill, Colin Hale, Kerensa Thorne, Justin Chiong, Susannah Condie, Henry Pertinez, Wendy Painter, Emma Wrixon, Lucy Johnson, Sara Yeats, Kim Mallard, Mike Radford, Keira Fines, Victoria Shaw, Andrew Owen, David G Lalloo, Michael Jacobs, Gareth Griffiths

**Affiliations:** 1 University of Liverpool, 70 Pembroke Place, Liverpool, UK; 2 Liverpool University Hospital NHS Foundation Trust, Prescot Road, Liverpool, UK; 3 Liverpool School of Tropical Medicine, Pembroke Place, Liverpool, UK; 4 Southampton Clinical Trials Unit, University of Southampton, Tremona Road, Southampton, UK; 5 University of Lancaster, Bailrigg, Lancaster, UK; 6 MRC Biostatistics Unit, University of Cambridge, Cambridge, UK; 7 Ridgeback Biotherapeutics, 3480 Main Highway, Miami, FL, USA; 8 Royal Free London NHS Foundation Trust, Pond Street, London, UK

## Abstract

**Objectives:**

AGILE is a Phase Ib/IIa platform for rapidly evaluating COVID-19 treatments. In this trial (NCT04746183) we evaluated the safety and optimal dose of molnupiravir in participants with early symptomatic infection.

**Methods:**

We undertook a dose-escalating, open-label, randomized-controlled (standard-of-care) Bayesian adaptive Phase I trial at the Royal Liverpool and Broadgreen Clinical Research Facility. Participants (adult outpatients with PCR-confirmed SARS-CoV-2 infection within 5 days of symptom onset) were randomized 2:1 in groups of 6 participants to 300, 600 and 800 mg doses of molnupiravir orally, twice daily for 5 days or control. A dose was judged unsafe if the probability of 30% or greater dose-limiting toxicity (the primary outcome) over controls was 25% or greater. Secondary outcomes included safety, clinical progression, pharmacokinetics and virological responses.

**Results:**

Of 103 participants screened, 18 participants were enrolled between 17 July and 30 October 2020. Molnupiravir was well tolerated at 300, 600 and 800 mg doses with no serious or severe adverse events. Overall, 4 of 4 (100%), 4 of 4 (100%) and 1 of 4 (25%) of the participants receiving 300, 600 and 800 mg molnupiravir, respectively, and 5 of 6 (83%) controls, had at least one adverse event, all of which were mild (≤grade 2). The probability of ≥30% excess toxicity over controls at 800 mg was estimated at 0.9%.

**Conclusions:**

Molnupiravir was safe and well tolerated; a dose of 800 mg twice daily for 5 days was recommended for Phase II evaluation.

## Introduction

In addition to life-saving therapies for COVID-19, there is an urgent need for effective antivirals, in order to reduce disease burden, prevent hospitalization and death and potentially decrease transmission of SARS-CoV-2. Since the natural history of infection is characterized by an early peak in viral load (within the first 5 days of infection),^1^ any antiviral would be expected to exert most effect when given early in the course of infection. AGILE is a randomized multi-arm, multi-dose, Phase Ib/IIa platform in the UK using a seamless Bayesian adaptive design^2^ to determine the safety, activity and optimal dose of multiple SARS-CoV-2 candidate therapeutics. Several candidates can be tested simultaneously (potentially sharing control group data) to increase efficiency.

We evaluated molnupiravir (EIDD-2801/MK-4482) for the treatment of COVID-19 in a seamless Phase I/II trial. Molnupiravir is rapidly and extensively converted (via host esterases) into the active ribonucleoside analogue β-d-N4-hydroxycytidine (NHC; EIDD-1931); cytochrome P450 enzymes are not a major route in the metabolism of molnupiravir and are not affected by the drug through enzyme induction or inhibition. Despite differences in model systems, the activity of molnupiravir has consistently been demonstrated *in vitro* and in animal models. In mice implanted with authentic human lung tissue, a prophylactic dose of 500 mg/kg given 12 h prior to inoculation with SARS-CoV-2 and every 12 h thereafter dramatically reduced viral pfu at 2 days post-inoculation.^3^ Furthermore, molnupiravir exerted an inhibitory effect on SARS-CoV-2 replication in the Syrian hamster model when commenced 12 h before or after experimental infection.^4^ Finally, molnupiravir significantly reduced viral titres in the nasal swabs and turbinate 4 days after infection in ferrets when given at 5 mg/kg twice daily initiated 12 h after inoculation or 15 mg/kg initiated 36 h after inoculation^5^ and was able to block transmission between ferrets. Current data warrant investigation of molnupiravir in human patients, including studies to define the appropriate dose for a human SARS-CoV-2 antiviral indication.

Molnupiravir has been evaluated in healthy volunteers in single (50–1600 mg) and multiple (50–800 mg for 5.5 days) ascending oral doses and was found to be well-tolerated.^6^ Preliminary data have also been presented from a study in patients with mild-to-moderate SARS-CoV-2 infection who received 200, 400 or 800 mg of molnupiravir twice daily for 5 days or placebo.^7^ Virus was cultured from nasopharyngeal swabs in only 42.9% of all PCR-positive patients at baseline and, of these, culture negativity was seen in all 47 evaluable subjects receiving molnupiravir (regardless of dose) versus 24% of subjects allocated to placebo.

Here we report Phase Ib results where we sought to determine the safety and tolerability of multiple ascending doses of molnupiravir in participants with PCR-confirmed SARS-CoV-2 infection who had symptoms within the preceding 5 days, which did not require hospitalization, to recommend a dose for Phase II. Secondary objectives included characterizing adverse events (AEs), serious AEs (SAEs), clinical outcomes (FLU-PRO, WHO Ordinal Scale, NEWS2 and mortality) and the pharmacokinetics of molnupiravir and its major metabolite EIDD-1931.

## Methods

### Study design, participants and ethics

This dose-escalation Phase I study (clinicaltrials.gov registration number NCT04746183) was designed as an open-label, randomized, controlled Bayesian adaptive trial in adult early infection in the community, coordinated by the National Institute for Health Research (NIHR) Southampton Clinical Trials Unit with participants recruited into the NIHR Royal Liverpool and Broadgreen Clinical Research Facility (UK). Eligible participants were men and women aged ≥18 years with PCR-confirmed SARS-CoV-2 infection who were within 5 days of symptom onset, free of uncontrolled chronic conditions and ambulant in the community with mild or moderate disease (see eligibility below). Women of childbearing potential and men were required to use two effective methods of contraception, one of which should be highly effective, throughout the study and for 50 and 100 days thereafter, respectively. Any of the following criteria excluded participants from the study: pregnant or breastfeeding women, stage 4 (severe) chronic kidney disease, clinically significant liver dysfunction, SpO2 <95% by oximetry or lung disease requiring supplementary oxygen, ALT and/or AST >5 times upper limit of normal, platelets <50 × 10^9^/L, experiencing any grade 3 or above Common Terminology Criteria for Adverse Events (CTCAE version 5), previously reported hepatitis C infection, known allergy to any study medication or having received any other experimental agents within 30 days of first dose of study drug (use of other co-medications was allowed). All participants provided written informed consent before enrolment. The study protocol was reviewed and approved by the UK Medicines and Healthcare product Regulatory Agency (MHRA) (EudraCT 2020–001860-27) and West Midlands Edgbaston Research Ethics Committee (20/WM/0136).

### Randomization and masking

Four sequential molnupiravir dosing tiers were defined *a priori* (300, 400, 600 and 800 mg twice daily for 5 days) with participants allocated using permuted blocks (block size 3, with no further stratification factors, generated by NIHR Southampton CTU statisticians) via MEDIDATA RAVE. Randomization used a 2:1 allocation ratio so that, within each cohort, 4 participants were randomly assigned to receive molnupiravir plus standard-of-care and 2 participants (controls) standard-of-care alone. This study was open label in accordance with conventional Phase I design, with a follow-on Phase IIa placebo-controlled study.

### Procedures

Participants with laboratory-confirmed SARS-CoV-2 infection or who had an illness compatible with COVID-19 (and who were subsequently confirmed to be positive) were screened against eligibility criteria, including presence and onset of symptoms within the previous 5 days. For safety reasons, in each cohort, the first participant randomized to molnupiravir (sentinel patient) was followed-up for 24 h before any subsequent participants were randomized. All participants who received molnupiravir received drug after at least a 2 h fasting period with a 4 h period of observation after the first dose.

We utilized a Bayesian adaptive design to accelerate decision making in this Phase I study. Briefly, we developed a dose–toxicity model,^8^ which estimates (as a continuous incremental probability) the ‘dose limiting toxicity’ (DLT) at day 7 of molnupiravir in controls and at each of the four predefined dosing tiers (300, 400, 600 and 800 mg twice daily)–see Figure 2. Tolerability was expressed as an excess in prevalence of DLTs of treatment over controls (who also suffered from symptoms of COVID-19 infection) with the model continually refined upon completion of each dosing cycle. Details are provided in Supplement S1 (available as Supplementary data at *JAC* Online). For each cohort, the Safety Review Committee (SRC) reviewed all available safety data, including at least 7 days of data for each participant in the cohort, and all accrued information on previous cohorts (up to a maximum follow-up of 28 days). This included AE data, vital signs data, ECG data and clinical laboratory evaluations, as well as any emerging data from other studies. Following SRC review, recommendations could be to de-escalate, escalate, remain at the same dose or continue to Phase II. A dose was deemed to be unsafe if the chance that treatment was associated with a 30% or higher risk of DLTs at day 7 was 25% or greater. The model recommended the next dose level according to which level was the most likely to correspond to an increase of 15%−25% in the DLT rate over control. However, the SRC made the ultimate decision whether to accept that the current dose was safe and to dose escalate and could decide to skip a dose if it did not more than double and was deemed safe by the Bayesian model. Once the dose-escalation Phase I was complete, the independent Data Monitoring and Ethics Committee reviewed data from the final SRC, along with their recommendations on the recommended Phase II dose, to ratify the recommended Phase 2 dose.

### Outcomes

The primary outcome was DLT using CTCAE version 5 (grades 3 and above) measured over 7 days and CTCAE grading related to platelets and/or lymphocytes, assessed in all participants, who were randomly assigned and received at least one dose of molnupiravir (unless randomized to control). Secondary outcomes for safety included AEs, SAEs, physical findings, vital signs and laboratory parameters, for pharmacokinetics included concentrations of molnupiravir and EIDD-1931 in plasma and for clinical included Patient Reported Outcome Measures (FLU-PRO), WHO COVID-19 Ordinal Scale (at days 15 and 29), NEWS2 (assessed during clinic days 15 and 29), mortality (days 15 and 29) and time from randomization to death (up to day 29).

### Pharmacokinetic sampling

Plasma was sampled at days 1 and 5 to measure the concentrations of molnupiravir and its major active metabolite EIDD-1931. On each sampling day, 2 mL of venous blood was collected pre-dose and at 30 min and 1, 2 and 4 h post-dose. All samples were rapidly cooled on wet ice and centrifuged (2000 **g** for 10 min) within 30 min of sample collection. Within 10 min of completing centrifugation, 150 mL of plasma was mixed with 450 μL of acetonitrile, vortexed and transferred to a −80°C freezer, prior to onward shipping for pharmacokinetic analyses. Drug concentrations were measured using a validated LC-MS/MS assay at Covance Clinical Laboratories, Leeds, UK.

Concentrations of EIDD-1931 in plasma on days 1 and 5 were described using summary statistics [geometric mean (90% CI), mean, SD, median and range] for each timepoint.

Key pharmacokinetic parameters, such as area under the concentration–time curve 0–4 h (AUC_0–4_), maximum concentration (*C*_max_) and time to *C*_max_ (*T*_max_), were determined by non-compartmental modelling methods (WinNonlin, Phoenix, v. 8.3, Pharsight, Mountain View, CA, USA) on days 1 and 5 for each dose and summarized descriptively. Accumulation ratios to day 5 were calculated for EIDD-1931 AUC_0–4_ and *C*_max_.

### Statistical analysis

All analyses are reported according to CONSORT 2010 and ICH E9 guidelines on Statistical Principles in Clinical Trials. All enrolled participants were included in both the evaluable population and the safety population for analysis.

The primary endpoint of DLTs up to 7 days after first dose were modelled using a Bayesian dose–toxicity model based on Mozgunov *et al*.^8^ The relationship between dose and toxicity was modelled using a two-parameter logistic model, where information can be shared across doses; in particular, the DLT rate in controls informs estimates for the active doses. The prior distributions for this model were calibrated to maximize the proportion of correct selection under a range of dose–toxicity scenarios where each dose considered in the study was the optimum one. The toxicity risk in controls was *a priori* assumed to be 10%. Further details are given in Supplement S1.

The dose–toxicity model was updated after every cohort of participants and the final model is presented as estimated DLT rates for each dose, alongside equal-tail 95% credible intervals. For active doses, we also present estimated additional toxicity above controls, the probability that the DLT rate falls within 15%–25% additional toxicity over controls (a predetermined acceptable target range for toxicity) and the probability of at least 30% additional toxicity over controls (deemed as unacceptably toxic). This is supported by the same information for up to day 29.

Baseline demographics are summarized within each dose (and controls) using descriptive statistics. Clinical endpoints are similarly summarized at days 15 and 29. The sample size was flexible, based on the need for the study to adapt to accruing safety data. Simulations were performed to assess model operating characteristics and to calibrate prior assumed four doses (plus controls), with cohorts of size six capped at a total of 30 participants.

Statistical analysis was undertaken in SAS version 9.4, STATA version 16 and R version 3.6.0.

## Results

**Figure 1.**
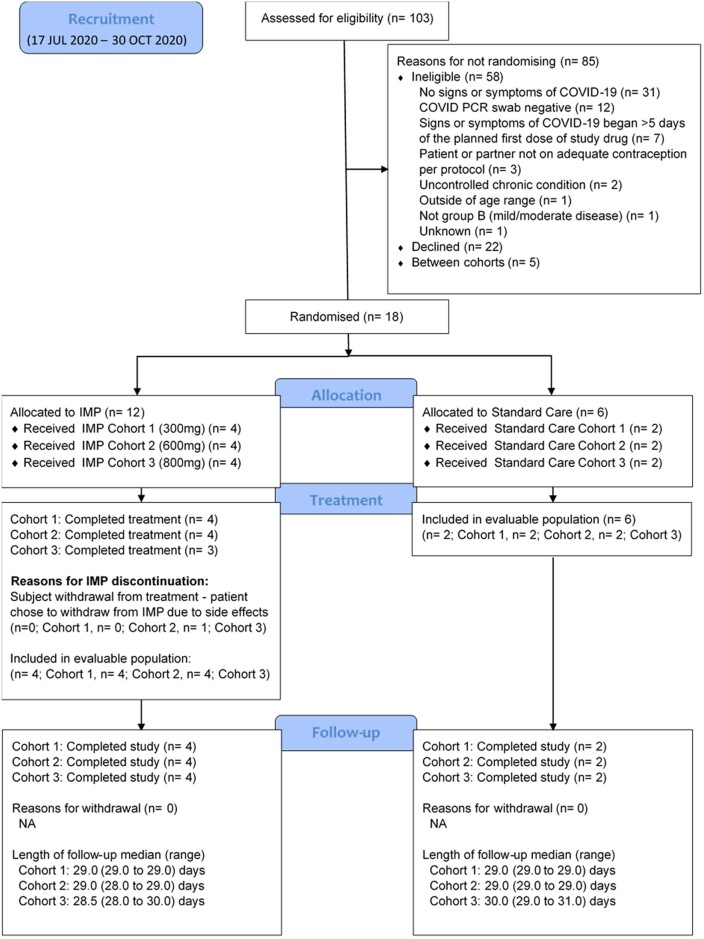
CONSORT diagram. IMP, investigational medicinal product; NA, not applicable. This figure appears in colour in the online version of *JAC* and in black and white in the print version of *JAC*.

Of 103 potential participants (Figure 1) who attended for screening, 58 were excluded (31 had no signs or symptoms of COVID-19, 12 tested negative for SARS-CoV-2 by PCR, 7 had signs or symptoms that began after 5 days of planned first dose, 2 had an uncontrolled comorbidity, 3 did not meet contraceptive requirements, 1 did not meet the age range, 1 did not meet the mild/moderate disease criterion, 22 declined, 5 were screened between dosing cohorts and 1 was unknown). Eligible individuals were randomly assigned within three sequential dose cohorts (300, 600 and 800 mg) of 6 participants each (i.e. a total of 18 participants within Phase I) and dosed in the period between 17 July 2020 and 30 October 2020. The baseline characteristics of participants were similar across all groups (Table 1) with an overall median age of 56 years, 72% (13/18) female and 33% (6/18) having a WHO COVID Ordinal Scale score of 1 (ambulatory mild disease). The median number of days (range) from symptom onset to randomization and treatment by the 18 participants was 4 (1–5).

**Table 1.** Baseline characteristics

	Molnupiravir			Standard-of-care total, *N *=* *6	Total, *N *=* *18
	300 mg, *N *=* *4	600 mg, *N *=* *4	800 mg, *N *=* *4		
Age at consent (years)					
*n*	4	4	4	6	18
median	56.0	43.0	39.0	59.0	56.0
range	51.0–80.0	22.0–60.0	25.0–63.0	22.0–63.0	22.0–80.0
Gender, *n* (%)					
male	1 (25.0)	2 (50.0)	0 (0.0)	2 (33.3)	5 (27.8)
female	3 (75.0)	2 (50.0)	4 (100)	4 (66.7)	13 (72.2)
Ethnicity, *n* (%)					
white—English/Welsh/Scottish/ Northern Irish	4 (100)	4 (100)	4 (100)	6 (100)	18 (100)
BMI (kg/m^2^)					
*n*	4	4	4	6	18
median	28.1	33.9	21.0	31.3	29.5
range	25.6–32.7	30.0–51.1	20.4–34.0	27.2–36.2	20.4–51.1
WHO score (day 1), *n* (%)					
1. ambulatory mild disease, asymptomatic; viral RNA detected	2 (50.0)	0 (0.0)	1 (25.0)	3 (50.0)	6 (33.3)
2. ambulatory mild disease, symptomatic; independent	1 (25.0)	4 (100)	3 (75.0)	3 (50.0)	11 (61.1)
3. ambulatory mild disease, symptomatic; assistance needed	1 (25.0)	0 (0.0)	0 (0.0)	0 (0.0)	1 (5.6)
WHO score (day 1)					
*n*	4	4	4	6	18
median	1.5	2	2	1.5	2
range	1–3	2–2	1–2	1–2	1–3
NEWS2 score (day 1)					
*n*	4	4	4	6	18
median	0	0	0.5	0	0
range	0–1	0–0	0–1	0–0	0–1
O_2_ saturation % (day 1)					
*n*	4	4	4	6	18
median	97.5	96.5	99.0	98.0	97.5
range	95.0–98.0	96.0–99.0	95.0–100.0	96.0–100.0	95.0–100.0
FLU-PRO total (day 1)					
*n*	3	4	4	6	17
median	0.7	0.8	1.0	0.6	
range	0.4–1.3	0.8–1.4	0.5–1.6	0.3–1.6	0.3–1.6
missing from electronic case record form, *n* (%)	1 (25.0)	0 (0.0)	0 (0.0)	0 (0.0)	1 (5.6)
Time from symptom onset to randomization (days)^a^					
*n*	4	4	4	6	18
median	4.0	4.0	3.5	4.0	4.0
range	3.0–4.0	4.0–4.0	2.0–4.0	1.0–5.0	1.0–5.0

Percentages are based on the number of patients in the study arm.

a Date of randomization is the same as date of first dose for all patients randomized to molnupiravir.

All molnupiravir participants received at least 1 dose with 3/4 (75%), 4/4 (100%) and 3/4 (75%) completing the full treatment in the 300, 600 and 800 mg cohorts, respectively. One participant on 300 mg twice daily only took 1 of 2 intended tablets for 2 of their treatment doses and one participant on 800 mg twice daily took only 2 doses on day 1, withdrawing from treatment for personal reasons unrelated to the study. The median number of molnupiravir doses received (range) was 10 (8–10), 10 (10–10) and 10 (2–10) and the median number of days on molnupiravir treatment (range) was 5.5 (5–6), 5 (5–5) and 5 (1–5) for the 300, 600 and 800 mg cohorts, respectively.

### Primary analysis

**Figure 2.**
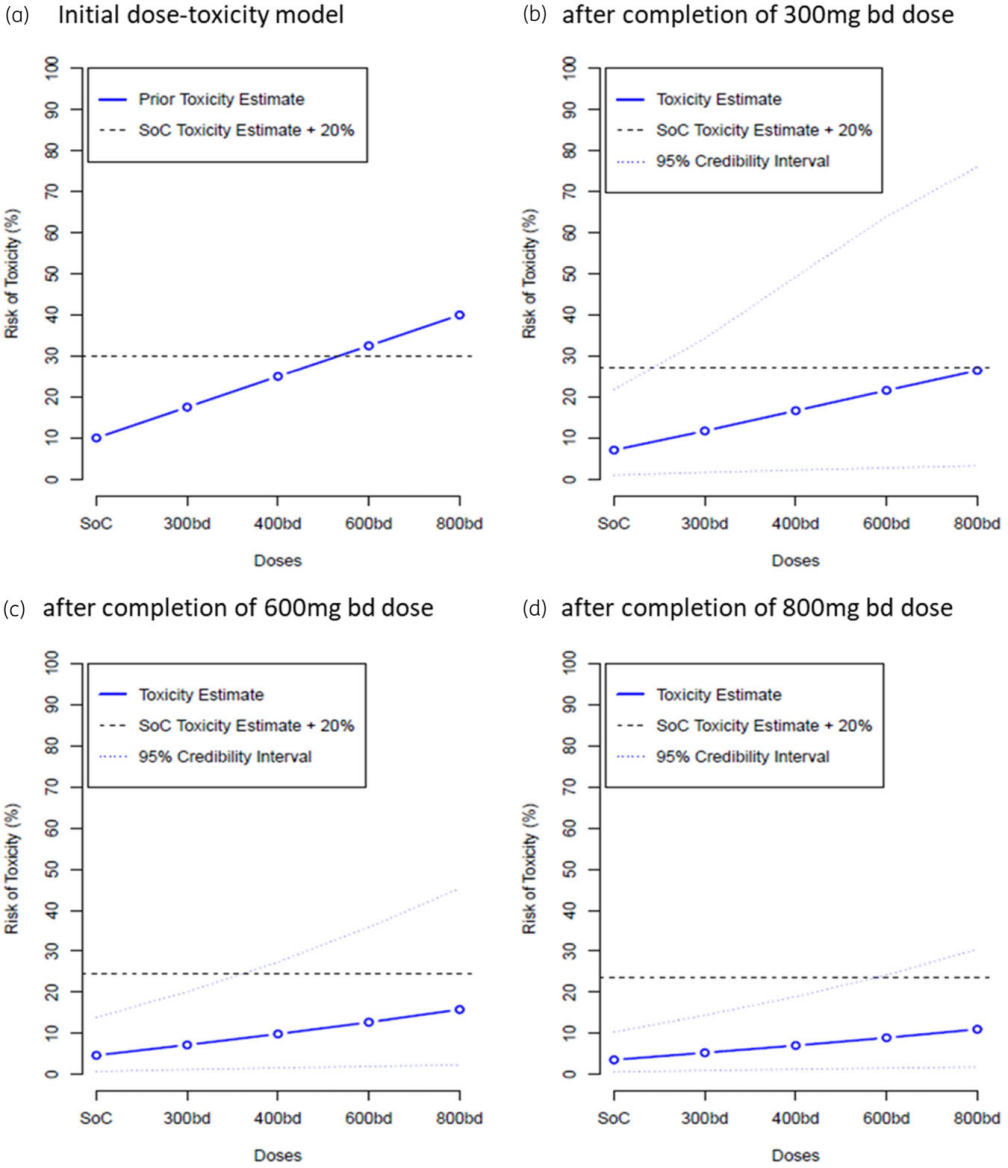
Primary endpoint - dose–toxicity plot up to day 7 (evaluable population). SoC, standard-of-care; bd, twice daily. This figure appears in colour in the online version of *JAC* and in black and white in the print version of *JAC*.

No participants in any cohort experienced a DLT or a grade 3 or above change in lymphocytes or platelets (for those with a normal baseline value) or a 2 or more grade increment in lymphocytes or platelets (for those with grade 2 or 3 at baseline). Following review by the SRC, dose cohort escalation went from 300 to 600 mg (skipping 400 mg) and then from 600 to 800 mg. Bayesian model DLT point estimates, 95% credible interval and the target toxicity level of 20% over the controls are shown in Figure 2. For data up to day 7, the maximum dose (800 mg) had an estimated DLT rate of 11.0% (equal-tail 95% credible interval of 1.8%–30.4%), with estimated 7.4% additional toxicity over controls and a probability of additional toxicity ≥30% over controls of 0.9%. As there were no DLTs recorded up to day 28, the results for day 7 are the same for day 28 and so are not repeated. These data support 800 mg twice daily as the recommended Phase II dose.

### Analysis of secondary endpoints

AEs were evenly distributed among the dose cohorts, including controls. Overall, 4 of 4 (100%), 4 of 4 (100%) and 1 of 4 (25%) of the participants receiving 300, 600 and 800 mg of molnupiravir, and 5 of 6 (83%) controls, had at least one AE, all of which were mild (≤grade 2). Molnupiravir was generally well-tolerated compared with controls and Table 2 describes the frequencies of events across the groups by system organ class and CTCAE term. No SAEs were reported. The most common symptoms were gastrointestinal (diarrhoea and nausea), respiratory (cough), CNS (loss of smell or taste) and flu-like symptoms.

**Table 2:** Overall toxicity summary by CTCAE version 5 term—safety population

Characteristic	Molnupiravir			Standard-of-care total, *N *=* *6
	300 mg, *N *=* *4	600 mg, *N *=* *4	800 mg, *N *=* *4	
Number of patients that experienced at least one AE, *n* (%)^a^	4 (100.0)	4 (100.0)	1 (25.0)	5 (83.3)
Summary of AEs, *n* (%)				
**cardiac disorders**	**0 (0.0)**	**0 (0.0)**	**1 (25.0)**	**0 (0.0)**
palpitations	0 (0.0)	0 (0.0)	1 (25.0)	0 (0.0)
**ear and labyrinth disorders**	**0 (0.0)**	**0 (0.0)**	**0 (0.0)**	**1 (16.7)**
tinnitus	0 (0.0)	0 (0.0)	0 (0.0)	1 (16.7)
**eye disorders**	**0 (0.0)**	**1 (25.0)**	**0 (0.0)**	**0 (0.0)**
blurred vision	0 (0.0)	1 (25.0)	0 (0.0)	0 (0.0)
**gastrointestinal disorders**	**2 (50.0)**	**3 (75.0)**	**0 (0.0)**	**2 (33.3)**
abdominal pain	1 (25.0)	0 (0.0)	0 (0.0)	0 (0.0)
diarrhoea	2 (50.0)	1 (25.0)	0 (0.0)	1 (16.7)
dyspepsia	0 (0.0)	1 (25.0)	0 (0.0)	0 (0.0)
nausea	1 (25.0)	2 (50.0)	0 (0.0)	1 (16.7)
oral dysesthesia	1 (25.0)	0 (0.0)	0 (0.0)	0 (0.0)
vomiting	1 (25.0)	0 (0.0)	0 (0.0)	0 (0.0)
**general disorders and administration site conditions**	**1 (25.0)**	**0 (0.0)**	**0 (0.0)**	**2 (33.3)**
fatigue	1 (25.0)	0 (0.0)	0 (0.0)	0 (0.0)
flu-like symptoms	0 (0.0)	0 (0.0)	0 (0.0)	2 (33.3)
non-cardiac chest pain	0 (0.0)	0 (0.0)	0 (0.0)	1 (16.7)
**infections and infestations**	**1 (25.0)**	**2 (50.0)**	**0 (0.0)**	**0 (0.0)**
herpes simplex reactivation	1 (25.0)	0 (0.0)	0 (0.0)	0 (0.0)
infections and infestations—other, specify^b^	0 (0.0)	1 (25.0)	0 (0.0)	0 (0.0)
thrush	0 (0.0)	1 (25.0)	0 (0.0)	0 (0.0)
**investigations**	**0 (0.0)**	**1 (25.0)**	**0 (0.0)**	**0 (0.0)**
ALT increased	0 (0.0)	1 (25.0)	0 (0.0)	0 (0.0)
GGT increased	0 (0.0)	1 (25.0)	0 (0.0)	0 (0.0)
**musculoskeletal and connective tissue disorders**	**1 (25.0)**	**0 (0.0)**	**0 (0.0)**	**1 (16.7)**
chest wall pain	1 (25.0)	0 (0.0)	0 (0.0)	0 (0.0)
myalgia	0 (0.0)	0 (0.0)	0 (0.0)	1 (16.7)
**nervous system disorders**	**2 (50.0)**	**1 (25.0)**	**0 (0.0)**	**2 (33.3)**
anosmia	1 (25.0)	0 (0.0)	0 (0.0)	0 (0.0)
dizziness	1 (25.0)	0 (0.0)	0 (0.0)	1 (16.7)
dysgeusia	1 (25.0)	0 (0.0)	0 (0.0)	1 (16.7)
headache	0 (0.0)	1 (25.0)	0 (0.0)	2 (33.3)
**psychiatric disorders**	**0 (0.0)**	**0 (0.0)**	**1 (25.0)**	**0 (0.0)**
anxiety	0 (0.0)	0 (0.0)	1 (25.0)	0 (0.0)
**renal and urinary disorders**	**1 (25.0)**	**0 (0.0)**	**0 (0.0)**	**1 (16.7)**
chronic kidney disease	0 (0.0)	0 (0.0)	0 (0.0)	1 (16.7)
urine discoloration	1 (25.0)	0 (0.0)	0 (0.0)	0 (0.0)
**respiratory, thoracic and mediastinal disorders**	**1 (25.0)**	**1 (25.0)**	**0 (0.0)**	**1 (16.7)**
cough	1 (25.0)	0 (0.0)	0 (0.0)	1 (16.7)
hoarseness	0 (0.0)	1 (25.0)	0 (0.0)	0 (0.0)
rhinorrhea	1 (25.0)	0 (0.0)	0 (0.0)	0 (0.0)
sore throat	0 (0.0)	1 (25.0)	0 (0.0)	0 (0.0)
**unclassified**	**1 (25.0)**	**1 (25.0)**	**1 (25.0)**	**0 (0.0)**
other—bilateral thigh pain	1 (25.0)	0 (0.0)	0 (0.0)	0 (0.0)
other—loose stools	0 (0.0)	0 (0.0)	1 (25.0)	0 (0.0)
other—worsening fatigue	0 (0.0)	1 (25.0)	0 (0.0)	0 (0.0)

Percentages are based on the number of patients in the study arm. CTCAE version 5 terms are used to classify AEs.

a All AEs were either grade 1 or 2.

b This AE reported in ‘other, specify’ free text field as ‘chest infection’.

At day 15, all participants had a WHO Ordinal Scale score of 1 or 2, with a median score (range) of 1.5 (1–2), 1.5 (1–2), 2 (2–2) and 1.5 (1–2) for 300, 600 and 800 mg of molnupiravir and controls, respectively. At day 15, 300 mg of molnupiravir, 600 mg of molnupiravir and controls had a median NEWS2 score of 0 (range 0−0), while 800 mg of molnupiravir had a median score of 1 (range 0−1). The median O_2_ saturation (range) was 97 (97–100), 97 (96–99), 99.5 (97–100) and 97 (96–99) for 300, 600 and 800 mg of molnupiravir and controls, respectively, with median FLU-PRO totals of 0.4 (0.2–10), 0.2 (0.1–0.6), 0.1 (0–0.3) and 0.2 (0–0.5), respectively (further details with comparable day 29 endpoints are provided in Supplement S2).

Virology samples were collected for future sequencing and characterization of variants; pharmacokinetic-pharmacodynamic analysis was not included in this Phase I analysis since sample sizes at each dose are too small to make inferences about antiviral activity.

### Pharmacokinetics

The prodrug molnupiravir was generally not detectable, or detected at low concentrations only at early timepoints (0.5 and 1 h post-dose), at all 3 doses (Table 3). Plasma concentrations of the nucleoside metabolite EIDD-1931 were detectable and showed no accumulation between days 1 and 5. At day 5, geometric mean NHC exposures (%CV) over the first 4 h of dosing (AUC_0–4_) for the 300 mg (*n *=* *4), 600 mg (*n *=* *4) and 800 mg (*n *=* *3) doses were 3470 (42.4), 3880 (56.3) and 7880 (39.0) ng·h/mL, respectively, with corresponding *C*_max_ values of 1620 (51.0), 1820 (84.6) and 4180 (28.1) ng/mL, respectively. *T*_max_ was 0.5–2.0 h.

**Table 3.** Geometric mean (%CV) pharmacokinetic parameters of EIDD-2801 and NHC following single- and multiple-dose administration of EIDD-2801

Parameter (units)	300 mg twice daily		600 mg twice daily		800 mg twice daily	
	day 1, *N *=* *4	day 5, *N *=* *4	day 1, *N *=* *4	day 5, *N *=* *4	day 1, *N *=* *4	day 5, *N *=* *3
Molnupiravir (EIDD-2801)						
AUC_0–4_ (ng·h/mL)	NC	NC	NC	NC	NC	NC
*C*_max_ (ng/mL)	5.76 (13.3)	NC	25.8	9.14^c^ (5.97–12.3)	8.43 (58.6)	7.79 (13.9)
*T*_max_^a^ (h)	0.500 (0.500–0.500)	NC	1.00^b^ (1.00–1.00)	0.500^c^ (0.500–0.500)	0.750 (0.500–1.00)	1.00 (1.00–1.00)
NHC (EIDD-1931)						
AUC_0–4_ (ng·h/mL)	3210 (40.5)	3470 (42.4)	4610 (33.7)	3880 (56.3)	9240 (41.0)	7880 (39.0)
*C*_max_ (ng/mL)	1490 (29.4)	1620 (51.0)	2230 (38.2)	1820 (84.6)	4440 (45.2)	4180 (28.1)
*T*_max_^a^ (h)	1.50 (1.00–2.00)	1.00 (1.00–2.00)	1.50 (1.00–2.00)	1.00 (1.00–2.00)	2.00 (1.00–2.00)	2.00 (1.00–2.00)

NC, not calculable.

a Median (min–max) presented.

b *n *=* *1 with quantifiable concentrations out of 4 subjects.

c *n *=* *2 with quantifiable concentrations out of 4 subjects.

## Discussion

To study the tolerability and safety of molnupiravir, we enrolled participants who presented within 5 days of symptoms, and who did not have severe disease, since we judged that the largest public health impact of this antiviral drug would be through deployment in the community for preventing hospitalization. In untreated SARS-CoV-2 infection, viral load peaks in the first week of illness,^1^ suggesting that early antiviral treatment may influence disease progression and potentially transmission.

We have established the safety and tolerability of molnupiravir in SARS-CoV-2 infected-individuals, alongside a conventional Phase I dose-ranging study in healthy volunteers (NCT04392219). We have shown that a dose of 800 mg of molnupiravir twice daily is safe and well-tolerated in participants with SARS CoV-2 infection; the plasma concentrations attained are within the target range based on scaling from animal models.^3^^,^^4^ AEs were commonly reported, affecting 9/12 and 5/6 participants on molnupiravir and controls, respectively. All were mild (grade 1–2) and included flu-like and upper respiratory symptoms, headache, myalgia, diarrhoea and nausea, which were also consistent with symptomatic COVID-19 disease.

AGILE utilizes complex innovative trial design methodology to accelerate early-phase evaluation of novel antiviral agents against SARS-CoV-2. Our Bayesian approach was selected to optimize statistical efficiency and to accelerate decision-making. Drug safety is not definitively established during Phase I and requires large numbers of individuals dosed in Phase III or IV. Rather, the AGILE design allowed us to establish (within an accelerated timescale) that a dose of 800 mg of molnupiravir twice daily for 5 days was sufficiently safe to progress into our continuation Phase IIa placebo-controlled trial (where safety continues to be monitored). Since full reproductive toxicological datasets were not available at the time of initiation, our study required stringent precautions to avoid pregnancy in participants or their partners.

To the best of our knowledge, this is the first published report describing the use of molnupiravir in SARS-CoV-2-infected individuals. We observed comparable exposures of EIDD-1931 to healthy volunteers^1^ and describe an approach for rapidly estimating a dose–toxicity relationship for Phase II evaluation. Whether or not molnupiravir will prove effective in treating COVID-19 will be determined in Phase II trials, which are currently underway, including our own, but the paucity of potent antiviral agents in the COVID-19 pipeline strongly argues for such accelerated approaches to early-phase drug development.

### Data sharing

The AGILE Trial Steering Committee will consider all reasonable requests by healthcare providers, investigators and researchers to provide anonymized data to address specific scientific or clinical objectives. The AGILE investigators are committed to reviewing requests from researchers for access to clinical trial protocols, de-identified patient-level clinical trial data and study-level clinical trial data.

## Supplementary Material

dkab318_Supplementary_Data

## Transparency declarations

W.H. is a cofounder, owner and advisor of/to Ridgeback Biotherapeutics LP. W.P. is employed by Ridgeback Biotherapeutics. S.H.K. has received research funding from ViiV Healthcare, Gilead Sciences and Merck for the Liverpool HIV Drug Interactions programme and for unrelated clinical studies. Unrelated to the current work, A.O. is a Director of Tandem Nano Ltd and co-inventor of patents relating to drug delivery. A.O. has received research funding from ViiV Healthcare, Merck and Janssen and consultancy from Gilead, ViiV and Merck not related to the current work. G.G. has received funding from Janssen-Cilag, AstraZeneca, Novartis, Astex, Roche, Heartflow, Celldex, BMS, BionTech, Cancer Research UK, NIHR, British Lung Foundation, Unitaid and GSK for unrelated academic clinical trials and programme funding. W.G. has received funding from the Wellcome Trust. All other authors: none to declare.

## Supplementary data

Supplement S1 and Supplement S2 are available as Supplementary data at *JAC* Online.
