# Supplementary material for: Optimal dose and safety of molnupiravir in patients with early SARS-CoV-2: a phase 1, open-label, dose-escalating, randomised controlled study
Source: J Antimicrob Chemother. Author manuscript; Available in PMC 2021 Nov 18. (PMC8598307; doi:10.1093/jac/dkab318)
Supplement: S1 [file EMS134033-supplement-S1.pdf]

Model-based dose-finding design

In this supplement we describe the details of the randomized model-based dose-finding design that has been used to aid the safety review committee in setting the dose for the subsequent cohort. The objective of this part of the trial is to study the safety of the drug for various doses. Safety is measured in terms of the risk of a dose-limiting toxicity (DLT) defined as any toxicity grade 3 or higher using CTCAE v5.0 over 7 days post randomization. The objective is to find the dose that corresponds to a 20% additional DLT risk over standard of care (SoC). This dose is referred to as the maximum tolerated dose (MTD). Patients are recruited in cohorts of 6 patients that are randomized 2:1 between an active dose of molnupiravir and SoC.

To describe the dose-toxicity relationship a Bayesian model-based dose-finding design based on Mozgunov et al. (2019) is used. The dose-toxicity relationship is modelled using a two-parameter logistic model. The toxicity risk at dose d_j, $j = 0, 1, 2, 3, 4$ is given by

$$p_j = \psi(\tilde{d}_j, \theta_1, \theta_2) = \frac{\exp(\theta_1 + \theta_2 \tilde{d}_j)}{1 + \exp(\theta_1 + \theta_2 \tilde{d}_j)} \tag{1}$$

where θ_1, θ_2 are unknown parameters with some prior distribution. The doses, $\tilde{d}_j$, are the standardized dose-levels (also known as a skeleton probabilities) obtained on the basis of prior estimates of the DLT probabilities, $\hat{p}_j^{(0)}$, on each dose. The standardized doses are found as $\tilde{d}_j = \frac{\text{logit}(\hat{p}_j^{(0)}) - \hat{\theta}_1^{(0)}}{\hat{\theta}_2^{(0)}}$ where $\hat{\theta}_1^{(0)}$, $\hat{\theta}_2^{(0)}$ are prior point estimates of the model parameters. Here, d_0 corresponds to the standard of care, and the standartised dose level $\tilde{d}_0$ is chosen such that $\tilde{d}_0 = 0$. This ensures that the estimate of dose-toxicity relationship, specifically, the slope θ_2, does not affect the DLT risk estimate on standard of care. The model with this skeleton is chosen as the dose level on standard of care, d_0, is not measured in the same units as the dose levels of the investigation (active) arm. At the same time, the inclusion of standard of care into the same model allows information to be borrowed from it, specifically, the fact that the DLT risk is greater at the investigational doses.

The prior distributions are defined as $(\theta_1, \log(\theta_2)) \sim \mathcal{N}(\mu, \Sigma)$ where $\mu = (\mu_1, \mu_2)^{\mathrm{T}}$ is the vector of means and

$$\Sigma = \begin{bmatrix} \sigma_1 & \sigma_{12} \\ \sigma_{12} & \sigma_2 \end{bmatrix}$$

The distribution of the unknown parameters θ_1, θ_2 are updated during the trial, and the allocation of a consecutive cohort is based on the update model.

To satisfy $\tilde{d}_0 = 0$, the prior distribution for the intercept parameter θ_1 should be chosen such that $\text{logit}(\hat{p}_0^{(0)}) = \hat{\theta}_1^{(0)}$ where $\hat{p}_0^{(0)}$ is the prior toxicity probability on SoC. Assuming 10% toxicity probability at the control arm, $\mu_1 = \text{logit}(0.1)$ was selected. The rest of the prior hyperparameters were calibrated using the scenarios given in Table 1 over a grid of values of each of them. Additionally, the standardized doses $\tilde{d}_j$ defined through the prior toxicity probabilities $\hat{p}_j^{(0)}$ are to be fixed prior to the trial. As there was no reliable information of the toxicity probabilties at the time of the planning in the COVID-19 population, these values were also calibrated.

The following parameters were found to result in good operating characteristics over all fours

scenarios: $\mu_2 = -0.05$ is the mean of the slope, covariance matrix

$$\Sigma = \begin{bmatrix} 1.10 & 0 \\ 0 & 0.30 \end{bmatrix},$$

and the difference in the toxicity probabilities between doses was found to be 0.075. The design proceeds as follows:

1. Cohorts of 6 patients are assigned to the first dose, and to standard of care, and DLT outcomes are evaluated;

2. Given the DLTs, the posterior distribution of the parameters θ_1, θ_2 are updated, and the induced posterior distribution of $p_j = p(d_j)$ is obtained.

3. The set of safe and admissible doses is found. The dose is deemed to be safe if $\mathbb{P}\left(p_j - p_0 \geq 0.3\right) < 0.25$, and is admissible if it corresponds to no more than doubling of the current dose.

4. The subsequent cohorts of patients is assigned to the safe, admissible dose level for which
$$\mathbb{P}\left(p_j - p_0 \in [0.15, 0.25]\right)$$
is maximised.

5. Steps 2-4 are repeated until the maximum number of patients is reached or all doses are deemed unsafe.

The proportion of each dose selection under 4 scenarios with $N = 30$ and assuming the toxicity probability at the SoC of 10% are given in Table 1.

Table 1: Proportion of each dose selection under the calibrated model for $N = 30$. The correct selections are in bold. Results are based on 2000 simulations.

	d_1	d_2	d_3	d_4
		Scenario 1		
Toxicity	**0.30**	0.45	0.60	0.70
Selection	**59.1%**	32.0%	5.7%	0.0%
		Scenario 2		
Toxicity	0.15	**0.30**	0.45	0.60
Selection	16.9%	**57.4%**	21.4%	3.8%
		Scenario 3		
Toxicity	0.12	0.15	**0.30**	0.45
Selection	2.8%	25.5%	**49.7%**	22.0%
		Scenario 4		
Toxicity	0.11	0.12	0.15	**0.30**
Selection	0.0 %	4.8%	28.9%	**65.9%**

It can be seen from Table 1 the proposed procedure results in a high proportion of correct selections across a wide range of scenarios.

References

Mozgunov, P., Jaki, T. and Paoletti, X. (2019) Randomized dose-escalation designs for drug combination cancer trials with immunotherapy. *Journal of Biopharmaceutical Statistics*, **29**, 359–377.
