# Supplementary material for: Optimal dose and safety of molnupiravir in patients with early SARS-CoV-2: a phase 1, open-label, dose-escalating, randomised controlled study
Source: J Antimicrob Chemother. Author manuscript; Available in PMC 2021 Nov 18. (PMC8598307; doi:10.1093/jac/dkab318)
Supplement: S2 [file EMS134033-supplement-S2.docx]

SUPPLEMENT S2:

Table. Clinical endpoints day 15 (Evaluable population)

|  | **MOLNUPIRAVIR**  **(300mg)**  **(n=4)** | **MOLNUPIRAVIR**  **(600mg)**  **(n=4)** | **MOLNUPIRAVIR**  **(800mg)**  **(n=4)** | **Standard Care**  **Total (n=6)** | **Total (n=18)** |
| --- | --- | --- | --- | --- | --- |
|  |  |  |  |  |  |
| WHO Score (day 15) – n (%) |  |  |  |  |  |
|  |  |  |  |  |  |
| 1. Ambulatory mild disease, asymptomatic; viral RNA detected | 2 (50·0%) | 2 (50·0%) | 0 (0·0%) | 3 (50·0%) | 7 (38·9%) |
| 2. Ambulatory mild disease, symptomatic; independent | 2 (50·0%) | 2 (50·0%) | 4 (100%) | 3 (50·0%) | 11 (61·1%) |
|  |  |  |  |  |  |
|  |  |  |  |  |  |
| WHO Score (day 15) |  |  |  |  |  |
| n | 4 | 4 | 4 | 6 | 18 |
| Median | 1·5 | 1·5 | 2 | 1·5 | 2 |
| Range | 1 to 2 | 1 to 2 | 2 to 2 | 1 to 2 | 1 to 2 |
|  |  |  |  |  |  |
|  |  |  |  |  |  |
| NEWS2 Score (day 15) |  |  |  |  |  |
| n | 4 | 4 | 4 | 6 | 18 |
| Median | 0 | 0 | 1 | 0 | 0 |
| Range | 0 to 0 | 0 to 0 | 0 to 1 | 0 to 0 | 0 to 1 |
|  |  |  |  |  |  |
|  |  |  |  |  |  |
| O_2_ Saturation (day 15) |  |  |  |  |  |
| n | 4 | 4 | 4 | 6 | 18 |
| Median | 97·0 | 97·0 | 99·5 | 97·0 | 97·0 |
| Range | 97·0 to 100·0 | 96·0 to 99·0 | 97·0 to 100·0 | 96·0 to 99·0 | 96·0 to 100·0 |
|  |  |  |  |  |  |
|  |  |  |  |  |  |
| FLU-PRO total (day 15) |  |  |  |  |  |
| n | 4 | 4 | 4 | 6 | 18 |
| Median | 0·4 | 0·2 | 0·1 | 0·2 | 0·2 |
| Range | 0·2 to 1·0 | 0·1 to 0·6 | 0·0 to 0·3 | 0·0 to 0·5 | 0·0 to 1·0 |
|  |  |  |  |  |  |

Note: Percentages are based on the number of patients in the study arm

Table: Clinical endpoints day 29 (Evaluable population)

|  | **MOLNUPIRAVIR**  **(300mg)**  **(n=4)** | **MOLNUPIRAVIR**  **(600mg)**  **(n=4)** | **MOLNUPIRAVIR**  **(800mg)**  **(n=4)** | **Standard Care**  **Total (n=6)** | **Total (n=18)** |
| --- | --- | --- | --- | --- | --- |
|  |  |  |  |  |  |
| WHO Score (day 29) – n (%) |  |  |  |  |  |
| 1. Ambulatory mild disease, asymptomatic; viral RNA detected | 3 (75·0%) | 2 (50·0%) | 3 (75·0%) | 5 (83·3%) | 13 (72·2%) |
| 2. Ambulatory mild disease, symptomatic; independent | 1 (25·0%) | 2 (50·0%) | 1 (25·0%) | 1 (16·7%) | 5 (27·8%) |
|  |  |  |  |  |  |
|  |  |  |  |  |  |
| WHO Score (day 29) |  |  |  |  |  |
| n | 4 | 4 | 4 | 6 | 18 |
| Median | 1 | 1.5 | 1 | 1 | 1 |
| Range | 1 to 2 | 1 to 2 | 1 to 2 | 1 to 2 | 1 to 2 |
|  |  |  |  |  |  |
|  |  |  |  |  |  |
| NEWS2 Score (day 29) |  |  |  |  |  |
| n | 4 | 4 | 4 | 6 | 18 |
| Median | 0 | 0 | 0.5 | 0 | 0 |
| Range | 0 to 1 | 0 to 1 | 0 to 1 | 0 to 1 | 0 to 1 |
|  |  |  |  |  |  |
|  |  |  |  |  |  |
| O_2_ Saturation (day 29) |  |  |  |  |  |
| n | 4 | 4 | 4 | 6 | 18 |
| Median | 98·5 | 97·5 | 99·0 | 97·5 | 98·0 |
| Range | 97·0 to 99·0 | 97·0 to 98·0 | 96·0 to 100·0 | 97·0 to 98·0 | 96·0 to 100·0 |
|  |  |  |  |  |  |
|  |  |  |  |  |  |
| FLU-PRO total (day 29) |  |  |  |  |  |
| n | 4 | 4 | 4 | 6 | 18 |
| Median | 0·3 | 0·1 | 0·1 | 0·2 | 0·1 |
| Range | 0·0 to 0·8 | 0·0 to 0·2 | 0·0 to 0·3 | 0·0 to 0·4 | 0·0 to 0·8 |
|  |  |  |  |  |  |

Note: Percentages are based on the number of patients in the study arm

*Reporting note: Mortality information at day 15 and day 29 (% died) will be included if any deaths occur up to day 29.*
